# Effect of V^IV^O(dipic-Cl)(H_2_O)_2_ on Lipid Metabolism Disorders in the Liver of STZ-Induced Diabetic Rats

**DOI:** 10.1155/2013/956737

**Published:** 2013-03-20

**Authors:** Fang Liu, Mingxia Xie, Deliang Chen, Jian Li, Wenjun Ding

**Affiliations:** College of Life Sciences, University of Chinese Academy of Sciences, No. 19A YuQuan Road, Beijing 100049, China

## Abstract

Vanadium complexes are potent antidiabetic agents for therapeutical treatment of diabetes. In the present study, we investigated the hypolipidemic effect of V^IV^O(dipic-Cl)(H_2_O)_2_ (V_4_dipic-Cl) in liver of streptozotocin- (STZ-)-induced diabetic rats. We found that diabetic animals exhibited hepatic inflammatory infiltration and impaired liver function along with triglyceride (TG) accumulation in the liver. V_4_dipic-Cl treatment not only ameliorated liver pathological state but also reduced hepatic TG level. Moreover, the upregulation of fatty acid translocase (FAT/CD36) mRNA (4.0-fold) and protein (8.2-fold) levels in the liver of diabetic rats were significantly reversed after V_4_dipic-Cl treatment. However, no significant effects of V_4_dipic-Cl on the mRNA expression of fatty acid metabolism-related fatty acid bounding protein 1 (FABP1) and fatty acid transporter 5 (FATP5) were observed. These results suggest that the modification of lipid metabolism-related FAT/CD36 in the liver of diabetic rats is likely involved in the hypolipidemic effects of V_4_dipic-Cl.

## 1. Introduction

Insulin dependent diabetes mellitus (IDDM), type 1 diabetes, is a form of diabetes mellitus that results from autoimmune destruction of insulin-producing *β*-cell of the pancreas. The deficiency or complete lack of insulin secretion leads to elevated blood glucose level [1, 2]. Patients with type 1 diabetes present lipid disorders or hyperlipidemia, including elevated levels of total serum cholesterol (TC), triglycerides (TG) [3], low-density lipoprotein (LDL-c), apolipoprotein A (ApoA), apolipoprotein B (ApoB) [4], malondialdehyde (MDA) [4, 5], very low-density lipoprotein (VLDL), and low level of high density lipoprotein (HDL-c) [6]. It is well known that the liver is a central organ in lipogenesis, gluconeogenesis, and cholesterol metabolism [7]. Hepatic lipid metabolism is influenced by the balance between the degradation and synthesis and/or import and export of triglyceride (TG) and fatty acids (FA). Fatty acids are important for many biological functions. Generally, fatty acids are degraded through *β*-oxidation or esterified and then stored as TG. Hepatic TG accumulation finally resulting in hepatic steatosis [8].Moreover, the FA transport process appears to be disturbed in obesity and diabetes mellitus [9].

Transport of unesterified FA into cells is a complex process involving protein catalysis [10]. Accumulating evidences proved that free fatty acids are taken up by the hepatocytes in a facilitated fashion rather than by passive processes [7, 11]. It is well known that fatty acid translocase is abundantly expressed in tissues with high metabolic capacity for fatty acids. A number of studies have shown that fatty acid translocase (FAT/CD36), fatty acid bounding protein (FABP), and fatty acid transporter (FATP) are membrane glycoproteins present on mononuclear phagocytes, adipocytes, and hepatocytes with multiple functions, which have also been identified to facilitate FA uptake and *β*-oxidation [12–15]. Several studies have demonstrated that FAT/CD36 as a shared transcriptional target is regulated by liver X receptor (LXR), pregnane X receptor (PXR), and aryl hydrocarbon receptor (AhR) [9, 16–18]. FA uptake into cells is regulated by altering the expressing of FAT/CD36 [9]. Overexpression of FAT/CD36 results in an increased rate of FA uptake and increased rate of FA metabolism [19]. Fatty acids are taken up into the cells and temporarily stored in a triglyceride pool. FA will be finally oxidized in mitochondria by means of carnitine palmitoyl transferase 1 (Cpt1) and peroxisomal acyl-coenzyme A oxidase 1 (ACOX1). 

The insulin-mimetic properties and antidiabetic effects of vanadium compounds have been widely documented both *in vivo* and *in vitro* [20–22]. Vanadium compounds stimulate glycogen synthesis [23] and lipogenesis [24] and inhibit lipolysis [24, 25]. Recently, various organic vanadium compounds with dipic, dipic-OH, or dipic-NH_2_ as organic ligand were reported as antidiabetic agents with little side effects and higher absorption than the simple salts [22, 26–28]. Moreover, it was observed that vanadate can restore the altered lipogenic enzyme activities to the normal level [29]. Our previous studies showed that vanadium compounds treatment potentially ameliorate lipid metabolism in diabetes [2, 22, 30–33]. However, the underlying mechanisms are not completely understood. Therefore, the aim of the study was an attempt to elucidate the hypolipidemic effect of V_4_dipic-Cl, if any, in regulating hepatic FAT/CD36-induced FA uptake and TG accumulation in STZ-induced diabetic rats. Moreover, serum biochemical parameters and histopathological examination were used to evaluate the side effect of V_4_dipic-Cl on hepatic functions in diabetic rats.

## 2. Materials and Methods

### 2.1. Chemicals

STZ was purchased from Sigma (Sigma-Aldrich, USA) H_2_dipic-Cl and V_4_dipic-Cl were gifts from Dr. Debbie C. Crans (Colorado State University, USA) [32]. Tissue triglyceride (TG) kit was purchased from Pplygen (Pplygen, China). RNA isolation reagent, UltraSYBR mixture, and *β*-actin antibody were purchased from Beijing CoWin Bioscience (China). RNA reverse transcription reagents were from Promega (USA). Radio immunoprecipitation assay lysis buffer (RIPA), HRP-labeled goat anti-rabbit IgG, HRP-labeled goat anti-mouse IgG, and electrochemiluminescence (ECL) reagent were purchased from Beyotime (China). FAT/CD36 antibody was from Santa (Santa Cruz, USA). All other chemicals used were of analytical grade.

### 2.2. Animals

Male Wistar rats (220 ± 10 g) were purchased from Beijing Academy of Military Medical Sciences. The animals were maintained under standard conditions (12 h light/dark cycle, 22 ± 2°C) and had free access to standard laboratory chow and water. The animals were cared for in accordance with the principles of the Guide for Care and Use of Experimental Animals.

### 2.3. Treatment Procedure

Diabetes was induced by a single intravenous injection of freshly prepared STZ (40 mg/mL; 55 mg/kg body weight) in 0.1 mol/L citrate buffer (pH 4.5). The control rats were only injected with an equal volume of citrate buffer. Animals with a fasting blood glucose level higher than 13.3 mM were considered to be diabetic rats. Normal and diabetic rats were randomly divided into four groups: Control group (C, *n* = 5), Diabetic group (D, *n* = 5), H_2_dipic-Cl-treated group (L, *n* = 5), and V_4_dipic-Cl-treated group (V4, *n* = 5). V_4_dipic-Cl was orally administrated to diabetic rats in drinking water at a concentration of 50 *μ*g V/mL daily for 28 days. We have selected this concentration of vanadium on the basis of earlier reports and the same has also been standardized in our laboratory to exhibit the glucose-lowering effects in STZ-induced diabetic animals [2, 30, 31, 33–36]. In this present study, fresh solutions of H_2_dipic-Cl and V_4_dipic-Cl were prepared every day and were given to the animals through drinking bottles.

### 2.4. Blood and Tissue Collection and Homogenate Preparation

 At the end of the treatment schedules, all animals were sacrificed. Blood was collected from the abdominal vein with a microsyringe. Serum was separated at 3,000 rpm for 15 min. The livers were perfused *in situ* with saline and then were immediately removed, collected, and stored in liquid nitrogen. Liver tissue homogenates were prepared in lysis buffer using an electric homogenizer.

### 2.5. Biochemical Analysis

 Biochemical parameters in serum, including TC, TG, HDL-c, LDL-c, alanine transaminase (ALT), and aspartate aminotransferase (AST) were determined using an OLYMPUS AU400 chemistry analyzer. Hepatic TG levels were measured by using tissue homogenates. The concentration of TG was determined using a tissue triglyceride assay kit.

### 2.6. Histological Examination

Sections measuring approximately 0.2 cm × 0.2 cm were taken from the liver of each rat. They were dehydrated through graded solutions of alcohol ending in two changes of absolute alcohol for 2 h each. They were cleared in 2 changes of xylene, infiltrated in 2 changes of paraffin wax for 2 h each, and embedded in molten paraffin wax. Sections were cut at 4 *μ*m with rotary microtome and stained with hematoxylin and eosin (H&E). Futher, the stained slides was observed under light microscope at 10x and 40x magnifications for histopathological examination.

### 2.7. Quantitative Analysis of Gene Expression

 Real-time PCR was carried out using the method described by Xue et al. [37]. Briefly, total RNA was extracted from the frozen liver by RNA Isolation Reagent. Then 1 *μ*g of total RNA was subjected to the reserve transcription reaction. The cDNA was used as a template to examine the mRNA levels of *FAT/CD36*, *FATP5*, *FABP1*, *Cpt1α*, *ACOX1*, *ApoB*, *LXR*, *PXR,* and *AhR* by using UltraSYBR mixture. *β*-*actin* was used as an internal control for normalization. The PCR cycle was as follows: initial denaturation at 95°C for 10 min, followed by 40 cycles of denaturation at 95°C for 30 s, annealing at 60°C for 30 s, and extension at 72°C for 30 s. The primers for target genes are shown in Table 1.

### 2.8. Western Blot

 Liver tissues were lysed in 1 ml of radio immunoprecipitation assay lysis buffer (RIPA) and then centrifuged at 14000 rpm for 5 min. Supernatants were collected and protein content was determined with protein assay kit. The lysates were subjected to sodium dodecyl sulfate polyacrylaminde gel electrophoresis (SDS-PAGE). The gels were transferred to polyvinylidene fluoride (PVDF) membrane by semidry electrophoretic transfer at an electric current 1 mA/cm^2^ for 90 min. The PVDF membrane was blocked with 5% no-fat milk for 1 h at the room temperature and then incubated with the primary antibody (1 : 1000) overnight at 4°C in a table concentrator. The membrane was washed per 5 min for 4 times prior to incubation in the secondary antibody (1 : 1000) solution for 1 h at the room temperature. Immunoreactive bands were detected with electrochemiluminescence (ECL) reagents according to the manufacturer's instructions. *β*-actin was included as a loading control.

### 2.9. Statistical Analysis

 Data are expressed as mean ± SEM. The statistical analysis was performed by one-way ANOVA followed by Tukey's test. Statistical significance was set at *P* < 0.05.

## 3. Results

### 3.1. Serum Parameters

**Figure 1:**
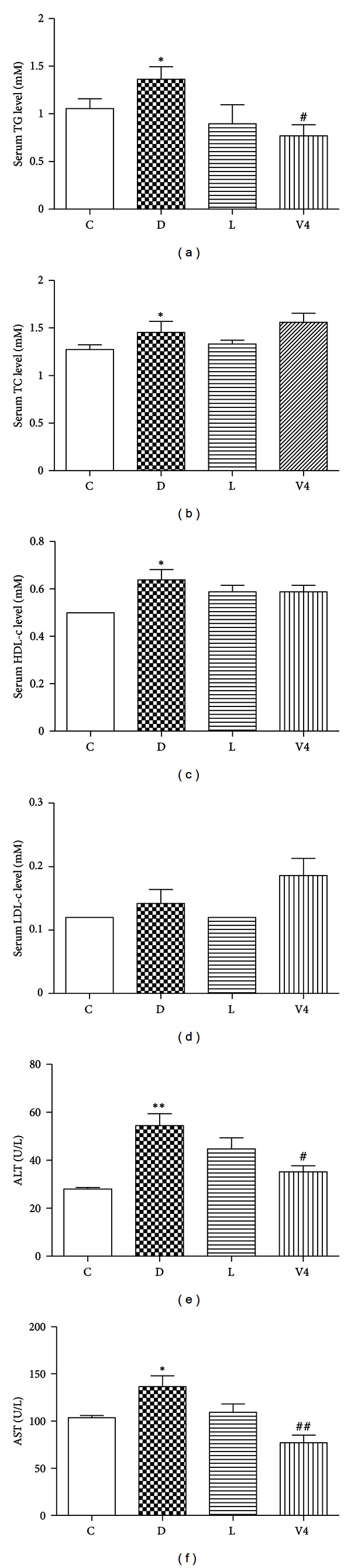
Effects of V_4_dipic-Cl on serum biochemical parameters in STZ-induced diabetic rats. (a) TG, (b) TC, (c) HDL-c, (d) LDL-c, (e) ALT, (f) AST. C: control group, D: diabetic group, L: H_2_dipic-Cl-treated group, V4: V_4_dipic-Cl-treated group. Values are expressed as mean ± SEM, *n* = 5. *P < 0.05, **P < 0.01 versus C. ^#^ *P* < 0.05, ^##^ *P* < 0.01 versus D.

 As shown in Figures 1(a)–1(c), serum TG, TC, and HDL-c levels in diabetic group were higher than those in control group. However, the level of serum TG was significantly decreased after treatment with V_4_dipic-Cl. The concentration of TC in diabetic rats remained unchanged after treatment with V_4_dipic-Cl. The LDL-c levels were not significantly different among the four groups of rats. Moreover, the activities of serum ALT and AST were markedly increased in diabetic rats. However, the ALT and AST activities were decreased in V_4_dipic-Cl-treated diabetic rats (Figures 1(e) and 1(f)).

### 3.2. TG Level and Histological Alteration in Liver

**Figure 2:**
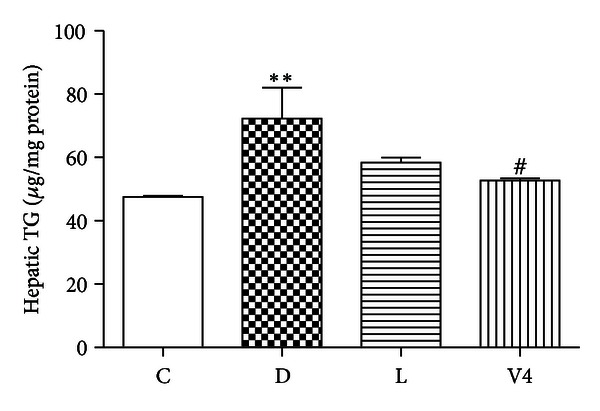
Effects of V_4_dipic-Cl on hepatic TG level in STZ-induced diabetic rats. C: control group, D: diabetic group, L: H_2_dipic-Cl-treated group, V4: V_4_dipic-Cl-treated group. Values are expressed as mean ± SEM, *n* = 3. **P < 0.01 versus C. ^#^ *P* < 0.05 versus D.

**Figure 3:**
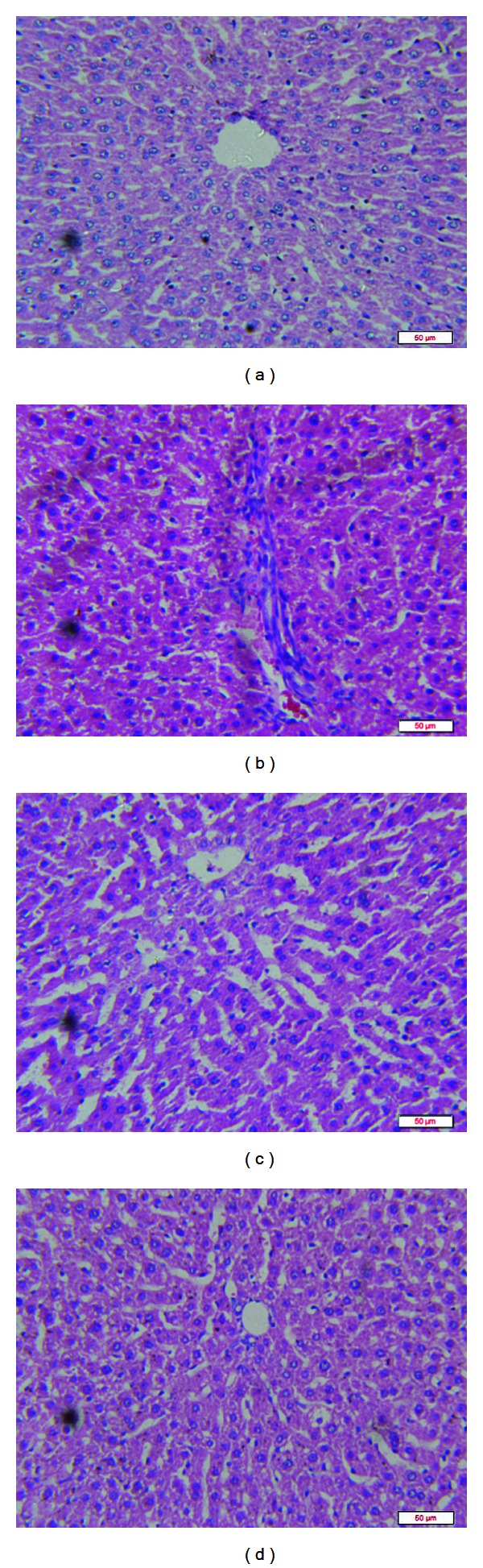
Effects of V_4_dipic-Cl on liver histological alterations in STZ-induced diabetic rats: (a) Control group, (b) Diabetic group, (c) H_2_dipic-Cl-treated group, (d) V_4_dipic-Cl-treated group (H&E, scale bar = 50 *μ*m, 400x).

The hepatic TG level in diabetic group was higher than that in normal rats, which was significantly decresed after treatment with V_4_dipic-Cl (Figure 2). In comparison with the control group (Figure 3(a)), the histological alterations were detected in the liver tissue of diabetic rats. Inflammatory cells infiltrate of liver lobules and dilated congested central vein were observed in Figure 3(b). However, the pathological alterations were ameliorated after treatment with V_4_dipic-Cl (Figure 3(d)) compared to those of H_2_dipic-Cl-treated diabetic rats (Figure 3(c)). 

### 3.3. Fatty Acids Transportation in Liver

**Figure 4:**
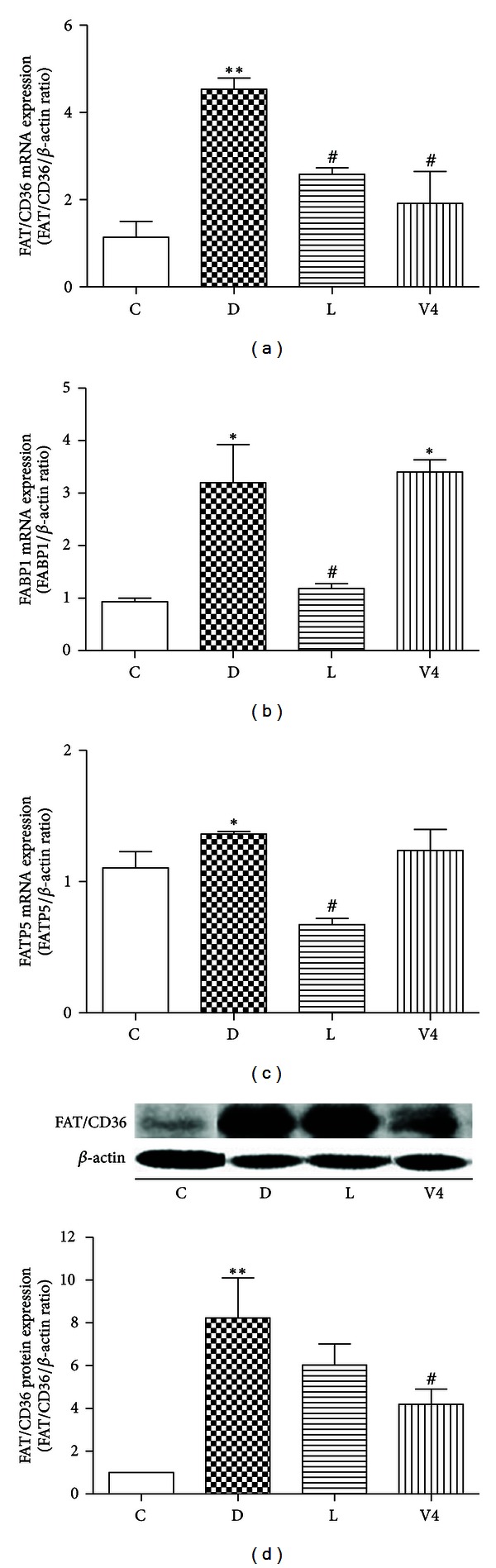
Effects of V_4_dipic-Cl on mRNA expression levels of* FAT/CD36, FABP1, and FATP5* and protein level of FAT/CD36 in STZ-induced diabetic rats. C: Control group, D: Diabetic group, L: H_2_dipic-Cl-treated group, V4: V_4_dipic-Cl-treated group. Values are expressed as mean ± SEM, *n* = 3. *P < 0.05, **P < 0.01 versus C. ^#^ *P* < 0.05, versus D.

 The mRNA expression levels of *FAT/CD36*, *FABP1,* and *FATP5* in diabetes group were higher than those in control group (Figure 4). However, the mRNA expression level of *FAT/CD36* was significantly decreased after treatment with V_4_dipic-Cl. Moreover, the mRNA expression levels of FAT/CD36, FABP1, and *FATP5* were significantly decreased in the H_2_dipic-Cl-treated group. In contrast, treatment with V_4_dipic-Cl did not affect the mRNA expression levels of *FABP* and *FATP* in STZ-induced diabetes.

### 3.4. Transcription Factors of FAT/CD36 in Liver

**Figure 5:**
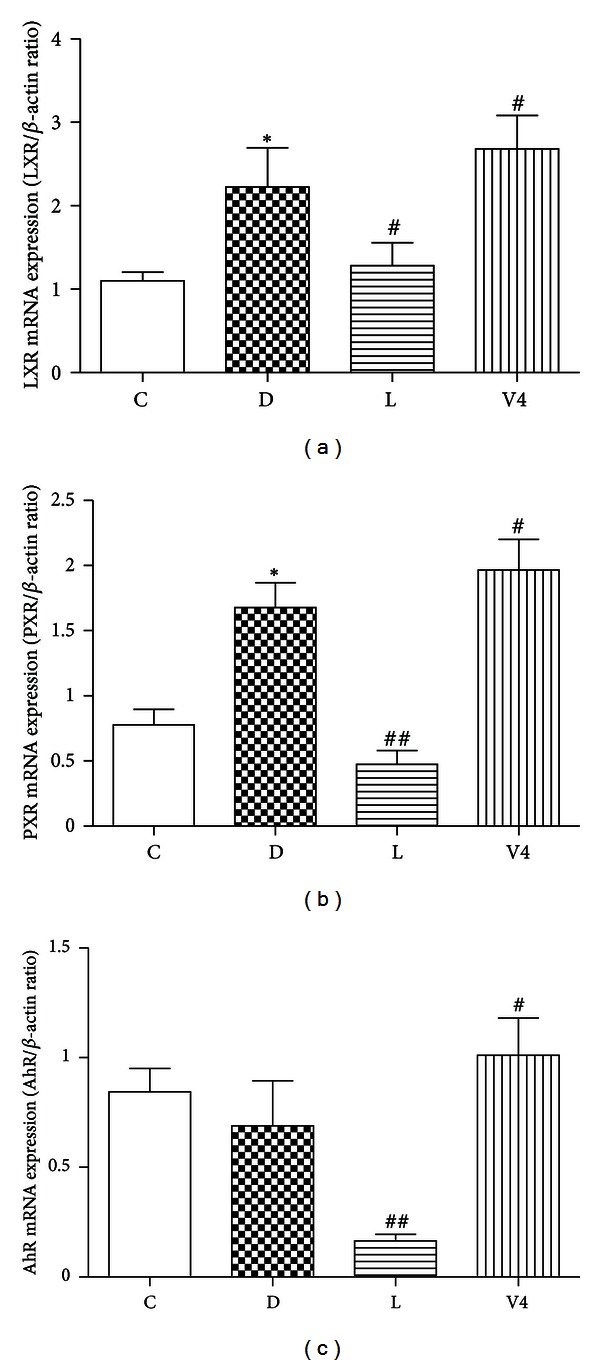
Effects of V_4_dipic-Cl on mRNA expression levels of *LXR*, *PXR,* and *AhR*. C: Control group, D: Diabetic group, L: H_2_dipic-Cl-treated group, V4: V_4_dipic-Cl-treated group. Values are expressed as mean ± SEM, *n* = 3. *P < 0.05 versus C. ^#^ *P* < 0.05 versus D, ^##^ *P* < 0.01 versus D.

The mRNA expression levels of *LXR* and *PXR* in diabetic group were significantly higher than those in control group. Moreover, the mRNA expression levels of *LXR*, *PXR,* and *AhR* were significantly elevated after treatment with V_4_dipic-Cl (Figure 5). However, the mRNA expression levels of *LXR*, *PXR,* and *AhR* were significantly decreased in the H_2_dipic-Cl-treated group as compared with the diabetic group (Figures 5(a)–5(c)).

### 3.5. Fatty Acids Oxidation in Liver

**Figure 6:**
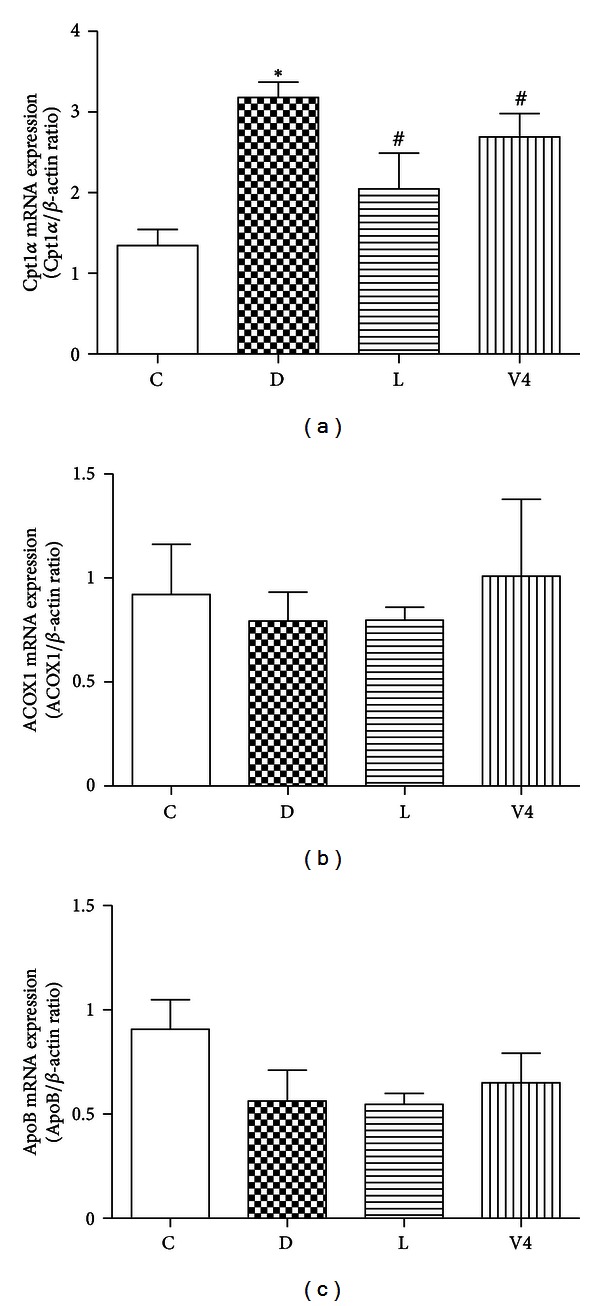
Effects of V_4_dipic-Cl on mRNA expression levels of *Cpt1α* *, ACOX1 *and* ApoB* in STZ-induced diabetic rats. C: Control group, D: Diabetic group, L: H_2_dipic-Cl-treated group, V4: V_4_dipic-Cl-treated group. Values are expressed as mean ± SEM, *n* = 3. *P < 0.05 versus C. ^#^ *P* < 0.05 versus D.

Carnitine palmitoyltransferase I*α*(Cpt1*α*) is located on the outer membrane of mitochondria and participates in fatty acid transportation into mitochondria. As shown in Figure 6(a), the mRNA expression level of *Cpt1α* was increased in diabetic group. However, the mRNA expression level of *Cpt1α* was significantly decreased after treatment with V_4_dipic-Cl and H_2_dipic-Cl. In contrast, the mRNA expression levels of *peroxisomal acyl-coenzyme A oxidase 1* (*ACOX1*) and *apolipoprotein B* (*ApoB*) were not significantly different among the four groups of rats (Figures 6(b) and 6(c)).

## 4. Discussion

Accumulating evidences have demonstrated that STZ-induced diabetes mellitus and insulin deficiency lead to hyperglycemia [38] and dyslipidemia [39]. It has been previously reported that hyperglycemia and dyslipidemia are associated with specific diabetic complications and disturbances in various tissues, such as diabetic nephropathy and cardiovascular diseases, but only limited data is available on the possible association between diabetic complications and liver function [30, 40]. The present study was designed to evaluate the effects of V_4_dipic-Cl on lipid metabolism disorders in the liver of STZ-induced diabetic rats.

It has been recognized that dyslipidemia is a frequent complication in all types of diabetes which can range from hypercholesterolemia to hyperlipoproteinemia [39]. Hyperlipidemia could be a factor for fatty liver formation [41]. In the present study, as expected serum TG and TC as well as hepatic TG levels were elevated in the diabetic group compared to those in the control group, which is consistent with other studies [3–5]. However, the elevated level of serum TG in diabetic rats was significantly decreased after treatment with V_4_dipic-Cl. This is in agreement with the evidence that vanadium compounds decrease the high levels of TG in serum and liver [29, 30]. Moreover, the altered expression of genes involved in lipid biosynthetic pathways in diabetes returned to normal level after treatment with vanadium compounds [2, 42]. 

Hyperglycemia is associated with liver dysfunction in IDDM [30, 41]. Elevated activities of serum aminotransferases are a common sign of liver diseases [30, 40]. Typical serum biochemical parameters, such as ALT and AST, are often examined to evaluate whether the liver is damaged or diseased. In the present study, our findings of elevated serum ALT and AST levels are in agreement with the findings of Zafar et al. [41]. The increase in ALT and AST activities may be due to the cellular damage in the liver caused by STZ-induced diabetes. After 28 days of treatment with V_4_dipic-Cl, the activities of both ALT and AST were significantly decreased. The result suggests that V_4_dipic-Cl may be capable of ameliorating the impaired liver function in STZ-induced diabetic rats, which is consistent with previously reported results for treatment with vanadium complexes [30].

Ohno et al. [43] described the fatty liver and hyperlipidemia in IDMM of treated shrews. In the present study, the histopathology of liver showed a development of the lesions which seems to be due to STZ treatment. Most liver sections showed inflammatory cells infiltrate of liver lobules and dilated congested central vein. These findings are in agreement with the findings of Degirmenci et al. and Zafar et al. who showed dilatation of veins and liver fibrosis in their study [41, 44]. However, we found that treatment with V_4_dipic-Cl dramatically improved pathologic lesions seen in the liver.

Free fatty acids are a major component of blood lipids and plays a key role in regulating blood lipid levels, especially in triglyceride metabolism [45]. In addition, elevated plasma FA is a risk factor for metabolic syndrome, which can lead to hyperlipidemia, fatty liver, and insulin resistance [46, 47]. FAT/CD36 is a rate-limiting enzyme in high-affinity peripheral FA uptake in the liver [48]. Thus, FAT/CD36 is an important regulator in the uptake of fatty acids in the liver and the pathogenesis of fatty liver disease. 

It was reported that FA uptake is reduced in FAT/CD36 null mice [49] and is reconstituted when FAT/CD36 is reexpressed [50]. Luiken et al. reported that *FAT/CD36* mRNA expression is increased in streptozotocin-induced diabetes [51]. Thus, FAT/CD36 may participate in the pathogenesis of liver ectopic fat deposition [16]. In the present study, we found that the mRNA expression level of *FAT/CD36* in diabetes group was significantly higher than that in control group. However, V_4_dipic-Cl treatment significantly resulted in decrease in the mRNA and protein expression levels of FAT/CD36 in V_4_dipic-Cl-treated group treatment. In addition to FAT/CD36, Goldberg and Ginsberg described that fatty acid-binding protein (FABP) and fatty acid transport protein (FATP) can mediate fatty acid uptake in the liver [52]. In the present study, the mRNA expression levels of FABP1 and FAFP5 were increased in diabetic group, which is consistent with the previous report that the expression of FABP is increased in STZ-induced diabetes [53]. However, treatment with V4dipic-Cl did not affect the expression of FABP and FATP in STZ-induced diabetes. Thus, we propose that V_4_dipic-Cl can mainly regulate fatty acid transporter FAT/CD36 in liver [54].

Zhou et al. described that nuclear acceptors AhR, PXR, and LXR cooperate to promote hepatic steatosis by increasing the expression of FAT/CD36 [16, 17]. More recently, Cheng et al. demonstrated that the mRNA expression level of *LXR* was markedly increased in diabetic rats [55]. Harano et al. reported that fenofibrate, PPAR*α* agonist, dramatically reduced hepatic triglyceride levels by activating expression of *ACOX1* and *Cpt1α* involved in fatty acid turnover [56]. In the present study, we also found that the mRNA expression levels of *LXR*, *PXR,* and *Cpt1α* were increased in diabetic group. However, treatment with V_4_dipic-Cl did not decrease the mRNA expression levels of *LXR*, *PXR,* and *AhR* as well as the FA oxidation-related Cpt1*α* and ACOX1 as compared to H_2_dipic-Cl-treated diabetic rats. It is possible that some other mechanisms may contribute to regulation of FAT/CD36 expression through modulating nuclear receptors by vanadium compounds. Our further research will focus on this. 

## 5. Conclusions

This study showed that V_4_dipic-Cl ameliorates STZ-induced hepatic inflammatory infiltration, liver disfunction, and hepatic TG accumulation. This effects were likely associated with the modification of lipid metabolism-related FAT/CD36 in liver. These results together with the previous observations suggest that V_4_dipic-Cl can be used as a therapeutic agent for treatment of metabolic disorder in diabetes mellitus. 

## Figures and Tables

**Table 1:** Primers for real-time PCR analysis.

Gene	Forward primer	Reverse primer
**β*-actin *	AAGATCATTGCTCCTCCTGAGC	CGTACTCCTGCTTGCTGATCCA
*FAT/CD36 *	AATCCTCTCCCTCTCTGGTGTC	CATGGCGAGGAACAGAACAT
*FABP1 *	CTTCTCCGGCAAGTACCAAGTG	CCCTTGATGTCCTTCCCTTTCT
*FATP5 *	CCTGCCAAGCTTCGTGCTAAT	GCTCATGTGATAGGATGGCTGG
*Cpt1*α**	GATCCACCATTCCACTCTGCTC	TGTGCCTGCTGTCCTTGATATG
*ACOX1 *	AGATTCAAGACAAAGCCGTCCA	TGATGCTCCCCTCAAGAAAGTC
*ApoB *	CAGCCAATAATGTGAGCCCCTA	TCCTATGCGCTTCCTGCTCTT
*LXR *	CCTATGTCTCCATCAACCACCC	ACTTGCTCTGAATGGACGCTG
*PXR *	GTGGAGCTAAAGAGCATGTGGC	TTCCTCCACACTTGGCATTTG
*AhR *	GCCAATACGCACCAAAAGCA	CCTGTTGGATCAAGGCACTCAT

*FAT/CD36*: fatty acid translocase*; FABP1*: fatty acid bounding protein1;* FATP5*: fatty acid transporter5;* Cpt1*α**: carnitine palmitoyltransferase I *α*;* ACOX1*: peroxisomal acyl-coenzyme A oxidase 1;* ApoB*: apolipoprotein B;* LXR*: liver X receptor;* PXR*: pregnane X receptor;* AhR*: aryl hydrocarbon receptor.
